# Exposure to Fine Particulate Matter Air Pollution Alters mRNA and miRNA Expression in Bone Marrow-Derived Endothelial Progenitor Cells from Mice

**DOI:** 10.3390/genes12071058

**Published:** 2021-07-10

**Authors:** Xiaohong Li, Petra Haberzettl, Daniel J. Conklin, Aruni Bhatnagar, Eric C. Rouchka, Mei Zhang, Timothy E. O’Toole

**Affiliations:** 1 Department of Anatomical Sciences and Neurobiology, University of Louisville, Louisville, KY 40202, USA; x0li0013@louisville.edu; 2 Kentucky Biomedical Research Infrastructure Network Bioinformatics Core, University of Louisville, Louisville, KY 40202, USA; eric.rouchka@louisville.edu; 3 Christina Lee Brown Envirome Institute, University of Louisville, Louisville, KY 40202, USA; petra.haberzettl@louisville.edu (P.H.); dj.conklin@louisville.edu (D.J.C.); aruni.bhatnagar@louisville.edu (A.B.); 4 Division of Environmental Medicine, University of Louisville, Louisville, KY 40202, USA; 5 Department of Computer Science and Engineering, University of Louisville, Louisville, KY 40202, USA; 6 Department of Medicine, University of Louisville Genomics Facility, Louisville, KY 40202, USA; mei.zhang@louisville.edu

**Keywords:** air pollution, endothelial progenitor cell, cardiovascular disease

## Abstract

Exposure to fine particulate matter (PM_2.5_) air pollution is associated with quantitative deficits of circulating endothelial progenitor cells (EPCs) in humans. Related exposures of mice to concentrated ambient PM_2.5_ (CAP) likewise reduces levels of circulating EPCs and induces defects in their proliferation and angiogenic potential as well. These changes in EPC number or function are predictive of larger cardiovascular dysfunction. To identify global, PM_2.5_-dependent mRNA and miRNA expression changes that may contribute to these defects, we performed a transcriptomic analysis of cells isolated from exposed mice. Compared with control samples, we identified 122 upregulated genes and 44 downregulated genes in EPCs derived from CAP-exposed animals. Functions most impacted by these gene expression changes included regulation of cell movement, cell and tissue development, and cellular assembly and organization. With respect to miRNA changes, we found that 55 were upregulated while 53 were downregulated in EPCs from CAP-exposed mice. The top functions impacted by these miRNA changes included cell movement, cell death and survival, cellular development, and cell growth and proliferation. A subset of these mRNA and miRNA changes were confirmed by qRT-PCR, including some reciprocal relationships. These results suggest that PM_2.5_-induced changes in gene expression may contribute to EPC dysfunction and that such changes may contribute to the adverse cardiovascular outcomes of air pollution exposure.

## 1. Introduction

Analysis of abundant epidemiological data has identified robust associations between exposure to fine particulate matter (PM_2.5_) air pollution and adverse health outcomes in general [1], and cardiovascular disease in particular [2,3]. These studies have linked acute exposure to PM_2.5_ with an increased incidence of myocardial infarction, stroke, cardiac arrhythmias, and sudden cardiac death [3,4,5], while chronic exposures appear to accelerate the progression of atherosclerotic disease [6,7]. Physiologically, these adverse cardiovascular outcomes are likely a consequence of a damaged or dysfunctional endothelium. Not only does the endothelial layer provide a physical separation between blood and surrounding tissue, it also plays an essential role in immune responses, thrombosis, and the control of blood pressure. Thus, the maintenance of vascular homeostasis is essential for overall health.

As with other tissues, the endothelium is subjected to physiological and environmental stimuli that affect its viability and functionality. Maintenance and repair of the endothelium are believed to be accomplished in part by a group of stem cells, the endothelial progenitor cells (EPCs) [8]. While their exact phenotypic identity, origin, and location are somewhat uncertain [9], upon hypoxic signals, these cells are mobilized from storage niches, home to a site of tissue damage, and participate in its repair through terminal differentiation or by paracrine stimulation of wound healing processes [10,11]. Supporting this reparative role, it has been found that EPC levels in peripheral blood increase after myocardial infarction and stoke, and that suppressed levels of these cells in circulation are associated with increased risk of cardiovascular disease [12,13,14], and can be predictive of major adverse cardiovascular events and death [15]. Thus, an accessible supply of these cells is essential to maintain endothelial and overall health. However, several studies have shown that the abundance and functionality of EPCs can also be impacted by environmental cues. Previous studies from our group have shown that exposure of a young healthy cohort to episodic increases of PM_2.5_ or exposure of mice to concentrated ambient fine PM_2.5_ (CAP) results in decreases of circulating EPCs [16,17]. Furthermore, bone marrow-derived EPCs cultured from mice exposed to CAP demonstrate defects in proliferation, in vitro tube formation, and vascular repair in vivo [18]. Likewise, exposure to ozone, metals, cigarette smoke, and cigarette smoke constituents have all been shown to negatively impact EPC number or function [19,20,21,22]. Consistent with the CAP-induced changes in proliferation and angiogenic potential we previously observed, we also detected changes in the expression of specific genes regulating these processes using a targeted approach [18]. However, the extent of PM_2.5_-induced gene expression changes in EPCs is unknown and it is consequently not clear what other cellular functions might be affected by exposure. Likewise, there is little information regarding PM_2.5_-induced changes in miRNA expression. Mice have been commonly used to study the acute and chronic effects of PM_2.5_ exposure and they appropriately model human outcomes. Thus, in the current study, to gain insight into PM_2.5_-induced genomic expression changes in EPCs and identify early biomarkers of cardiovascular disease initiation or progression, we performed a global transcriptomic analysis of exposed mice. Our results identified differentially expressed mRNAs and miRNAs and those cellular functions impacted by these changes, further clarifying the mechanistic basis of PM_2.5_-induced cardiovascular disease.

## 2. Materials and Methods

### 2.1. Mice, Exposures, and RNA Extraction

**Figure 1:**
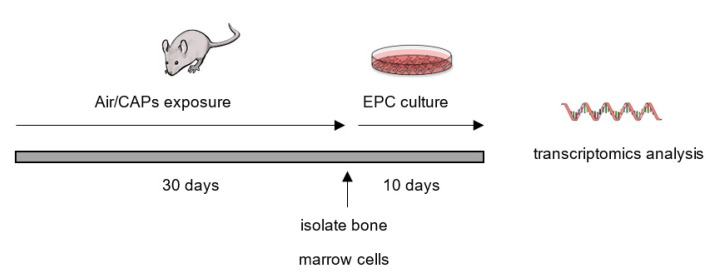
Experimental protocol. Illustrated is the timeline of animal treatment, cell culture, and RNA analysis.

Male C57BL/6J mice were obtained at 11 weeks of age from Jackson Laboratories and acclimated for 1 week in pathogen-free housing conditions. The animals were kept on a 12 h light/dark schedule and were supplied with food and water *ad libitum*. Mice (*n* = 3; 1 cage) were randomly selected and exposed to CAP generated by a Versatile Aerosol Concentration and Enrichment System (VACES) in the Inhalation Facility of the University of Louisville, as previously described [16,17]. Mice exposed to HEPA-filtered air (*n* = 3; 1 cage) were used as controls. Exposures to CAP or filtered air were 6 h per day (7 a.m. to 1 p.m.) for 30 consecutive days, and over this time course, the average CAP level was 67 μg/m^3^. These animals were monitored daily by veterinarian staff for well-being. The animals were euthanized (pentobarbitol injection (50 mg/kg, i.p., and exsanguination)) immediately after the final exposure (CAP- and air-exposed animals intermingled) and bone marrow cells isolated and EPCs cultured as previously described [18]. After 10 days in culture, the cells were harvested, and total RNA was isolated using the miRNeasy isolation kit (Qiagen). RNA quality was confirmed using an Agilent Bioanalyzer. A schematic of the experimental protocol illustrated in Figure 1.

### 2.2. Next-Generation mRNA Sequencing

#### 2.2.1. Library Preparation

Libraries were prepared using the TruSeq Stranded mRNA Library Prep kit (20020594, Illumina, San Diego, CA, USA). In brief, poly-A enrichment of RNA samples (250 ng) was accomplished in two purification steps using oligo dT beads. Purified mRNA was chemically fragmented through a heated digestion with divalent cations. Eluted samples were then used for first- and second-strand cDNA synthesis, and the resulting cDNA was isolated using Agencourt AMPure XP Beads (A63881, Beckman Coulter Life Sciences, Indianapolis, IN, USA). After adenylation of the 3′ end to prevent blunt end fragments from ligating to each other, dual-index adapters were ligated, and the samples were again purified with the same beads. Adapter ligated DNA fragments were then enriched and amplified with PCR to create the final cDNA libraries. The concentrations of these libraries were measured by Qubit dsDNA HS Assay Kit (Q32851, Invitrogen, Waltham, MA, USA), and their quality was assessed using the Agilent Bioanalyzer.

#### 2.2.2. Sequencing

Similar molar amounts were pooled, and their quantity and quality were assessed using the MiSeq Reagent Nano Kit V2 (MS-103-1001, Illumina). The libraries and PhIX control (FC-110-3001, Illumina) were denatured and diluted using the manufacturer’s directions and sequenced on an Illumina MiSeq. On the basis of the sequencing behavior of the libraries compared to PhIX, we re-pooled, denatured, and diluted equal amounts of the libraries, and 1.8 pM was loaded on the NextSeq with 1% PhIX spike-in. After the first sequencing run, the libraries were re-pooled for a second run with the goal of having an equal number of reads per sample after results from both runs were combined, which gave an average of 35 million reads per sample. All sequencing was performed at the University of Louisville Genomics Facility on the Illumina NextSeq 500 using the NextSeq 500/550 75 cycle High Output Kit v2 (FC-404-2002, Illumina).

#### 2.2.3. Data Analysis

The 48 fastq single-end raw sequencing files representing the air and CAP conditions with three biological replicates and eight lances per replicate were downloaded from Illumina’s BaseSpace (https://basespace.illumina.com/, accessed on 13 December 2019). The eight single-end raw fastq files across 8 sequencing lanes for each replicate designated L001 through L008 in the file names from the sequencing run were concatenated into one single-end fastq file using the unix cat command. Quality control (QC) of the raw sequence data was performed using FastQC (version 0.10.1). The FastQC results showed the sequences for all the samples had a Phred score above 30, which indicated 99.9% accuracy in base calling and were considered to be good quality. Therefore, sequence trimming was not performed. The concatenated sequence samples were directly aligned to the *Mus musculus* reference genome assembly (mm10.fa) using STAR (version 2.6) [23]. The alignment rate ranged from 99.12 to 99.98 percent across the samples. For the identification of differentially expressed genes (DEG) using DESeq2 [24], the raw reads counts were extracted from the STAR-aligned bam format files using HTSeq version 0.10.0 and the *Mus musculus* gtf file (mm10.gtf). Supplementary Table S1 indicates the number of raw reads and number of reads successfully aligned for each of the six samples. The raw read counts for six samples being normalized using the relative log expression (RLE) method and then filtered to exclude genes with fewer than 10 counts across the samples with the aid of *DESeq2*. DEGs (CAP versus air) were identified and a Benjemini–Hochberg adjusted *p*-value (*q*-value) cutoff ≤0.05 with log_2_ fold change (FC) ≥0.6 was used to define differential expression. RNAseq data are available at GEO accession number GSE153038.

### 2.3. Next-Generation miRNA Sequencing

#### 2.3.1. Library Preparation

Libraries were prepared with 100 ng from each of the RNA samples using the QIAseq miRNA Library Kit (331502, Qiagen, Beverly, MA, USA) following the manufacturer’s instructions. Briefly, specially designed 3′ and 5′ adapters were ligated to mature miRNAs. The ligated miRNAs were then reverse-transcribed to cDNA and purified with magnetic beads (QIAseq miRNA NGS beads). The eluted cDNA was amplified with a universal forward primer and a sample indexing reverse primer. The amplified libraries were again purified with magnetic beads to remove adapter dimers and unwanted RNA species. Library quantity and quality were analyzed on an Agilent Bioanalyzer using the Agilent high sensitivity DNA Kit (5067-4626, Agilent Technologies, Santa Clara, CA, USA).

#### 2.3.2. Sequencing

Similar molar amounts of the libraries were pooled and run on MiSeq to test for quantity and quality. Then, the libraries and PhIX control (FC-110-3001, Illumina) were denatured and diluted following manufacturer’s directions, and 300 μL of each were combined and sequenced on Illumina MiSeq. The concentration of the libraries was corrected and the libraries re-pooled on the basis of the results from the initial MiSeq run. After denaturation and dilution, sequencing was performed on the University of Louisville Genomics Facility’s Illumina NextSeq 500 using the NextSeq 500/550 75 cycle High Output Kit v2 (FC-404-2002, Illumina).

#### 2.3.3. Data Analysis

The 48 fastq single-end raw sequencing files representing the air and CAP conditions with three biological replicates and eight lances per replicate were downloaded from Illumina’s BaseSpace (https://basespace.illumina.com/, accessed on 3 January 2020). The eight single-end raw sequencing files (fastq) for each replicate designated L001 through L008 from the sequencing run were concatenated into one single-end fastq file using the unix cat command. Quality control (QC) of the raw sequence data was performed using FastQC (version 0.10.1). The FastQC results indicated quality trimming was unnecessary since the minimum quality value of a Phred score for all samples was well above 30 (1 per 1000 error rate). Given this, preliminary adapter trimming was performed on each of the samples to remove the Qiagen 3′ and 5′ adapter sequence with Trimmomatric v0.33 [25]. For all of the samples, a unique peak around 22 bp was identified that indicated that little if any other non-coding RNAs were selected in the library processing. As expected, this peak represented the length of the mature miRNAs. The data were further analyzed using mirDeep2 [26] and DESeq2 [24]. Briefly, the trimmed sequence reads were directly mapped to the mouse mm10 reference genome assembly using mirDeep2 package (v0.07) implementing bowtie mapper (v1.1.1) [27]. Supplementary Table S2 indicates the number of raw reads, number of reads after trimming, and number of reads successfully aligned for each of the six samples. The aligned sequences were then used as inputs into mirDeep2 along with the latest release of the mirBase version 22 database (v22) containing mature miRNA and miRNA hairpin sequences. The result was a file containing the number of reads mapping to each of the 2061 mouse miRNAs. After quantification, the resulting counts for each miRNA in each sample were combined into a reads matrix. Using the counts table resulting from the previous step, we determined the differentially expressed miRNAs (*q* ≤ 0.05; upregulated: log_2_FC ≥ 0.6; downregulated: log_2_FC < −0.6) using DESeq2. miRNA-seq data are available at GEO accession GSE153038.

### 2.4. In Silico INGENUITY Network Analysis

Canonical pathway and biological function analysis of DEGs and differentially expressed miRNAs (DE miRNAs) was performed using Ingenuity Pathway Analysis (202 Qiagen version 57662101).

### 2.5. qRT-PCR

Quantitative RT-PCR (qRT-PCR) of selected mRNA species was performed by initial, first-strand cDNA synthesis (High Capacity cDNA Reverse Transcription Kit, Thermo-Fisher), followed by real-time PCR using specific primers as recommended by the manufacturer (Supplementary Table S3: TaqMan gene expression assays, Thermo-Fisher). Quantitative RT-PCR of selected miRNA species was performed using specific TaqMan MicroRNA assays (Supplementary Table S3; Thermo-Fisher). All amplifications were performed on a Quant Studio 5 thermal cycler (Applied Biosystems). The internal controls used were *Hprt* for mRNAs and *sno202* for miRNAs. Fold changes were calculated using the Ct method [28] and were reported with a standard error.

## 3. Results

### 3.1. mRNA Analysis

**Figure 2:**
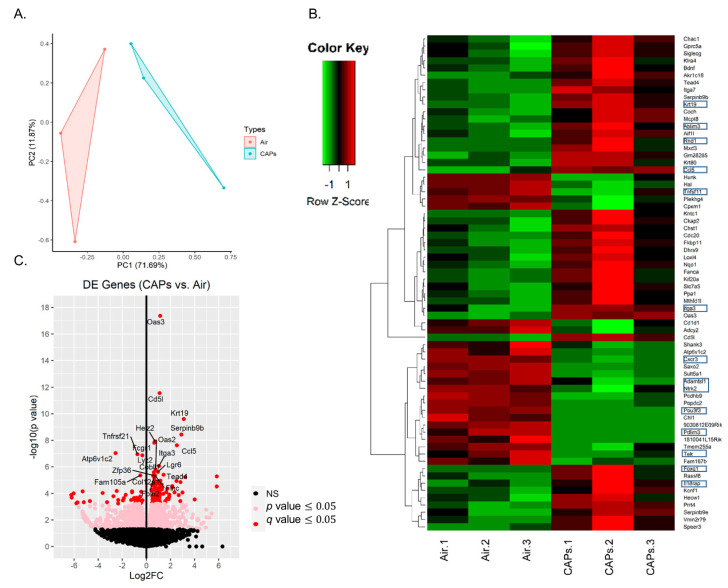
mRNA sequence analysis. Illustrated is a principal component analysis (PCA) for the analysis of mRNA (**A**), a heat map depicting 68 differentially expressed genes (|log_2_FC| ≥ 1), where the Z-score values obtained by scaling the expression of the genes is denoted in a color code from lower (green) to higher (red) (**B**), and a volcano plot (log_2_FC vs. −log10 (*p*-value)) of DEGs (**C**). Boxed genes (**B**) are those that are implicated in EPC function.

To evaluate similarities and differences in mRNA expression between the air and CAP groups, we initially performed a principal component analysis (PCA) on the relative log expression (RLE)-normalized counts of the six samples for all genes. In this analysis (Figure 2A), the two principal components (PC1 and PC2) represent two-dimensional data with 71.7% and 11.9% variation in the six mRNA samples, respectively. The PC1 axis (the first principal direction with all six mRNA samples) showed the largest variation, which explained most of the variance in the original data. The PC2 axis is the second most important direction and orthogonal to the PC1 axis. For these samples, the CAP group (blue) was partially separated from the air group (red), and some variation within the groups was observed.

To identify transcriptional alterations driven by CAP exposure, we performed mRNA expression profiling. Hierarchical clustering of the expression of 68 differentially expressed genes (DEGs) revealed a distinct transcriptomic profiling pattern between the samples as illustrated in a heatmap (Figure 2B) from low expression (green) to high expression (red). Given a *q*-value cutoff ≤ 0.05, we identified 166 genes that were differentially expressed. This included 122 genes that were upregulated and 44 that were downregulated in the CAP vs. air groups. The most upregulated or downregulated DEGs are located in a volcano plot (*p*-value vs. FC) in Figure 2C. We further analyzed these DEGs according to a fold change. Given a *q*-value ≤ 0.05, we identified 104 that were upregulated by >1.5 fold (log_2_ FC ≥ 0.6). The top 20 of these genes are listed in Table 1, while the complete list is provided in Supplementary Table S4. Given a *q*-value ≤ 0.05, we identified 34 that were downregulated by <−1.5 fold (log_2_FC ≤ −0.6). The top 20 of these genes are listed in Table 2, while the complete list is provided in Supplementary Table S5.

### 3.2. miRNA Analysis

**Figure 3:**
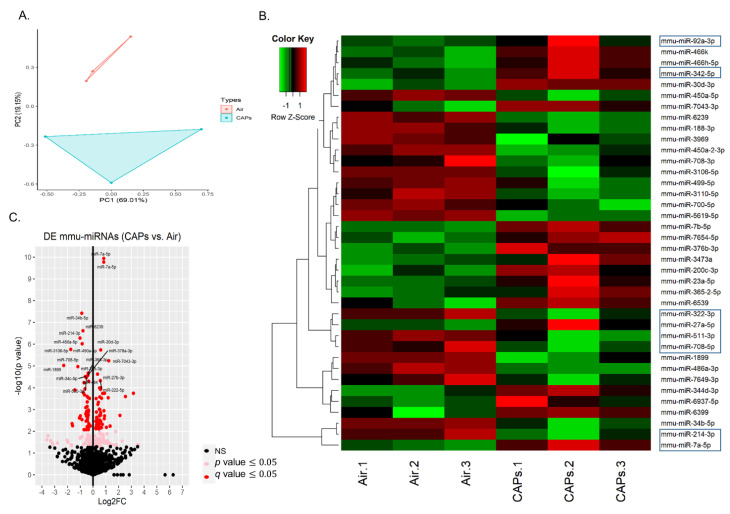
miRNA sequence analysis. Illustrated is a principal component analysis (PCA) for the analysis of miRNA (**A**), a heat map depicting 38 differentially expressed miRNAs (|log_2_FC| ≥ 0.6 where the Z-score values obtained from scaling the expression of the miRNAs is denoted in a color code from lower (green) to higher (red) (**B**), and a volcano plot (log_2_FC vs. −≤log10 (*p*-value)) of the DE miRNAs (**C**). Boxed miRNAs (**B**) are those that are implicated in EPC function.

Using the same samples, we performed a similar analysis of miRNA expression. PCA analysis of these samples (Figure 3A) revealed 69.0% and 19.2% variation of the six miRNA samples. This analysis reveals clear separations between the CAP (blue) and air (red) samples. Hierarchical clustering of the expression of 38 differentially expressed miRNAs (DE miRNAs) revealed a unique miRNA profiling pattern, as illustrated in a heatmap (Figure 3B). Given a *q*-value cutoff ≤ 0.05, we identified 108 unique miRNAs that were differentially expressed. This included 55 miRNAs that were upregulated and 53 that were downregulated in the CAP vs. air groups. The most upregulated and downregulated miRNAs are located in a volcano plot (Figure 3C). We further analyzed these DE miRNAs according to fold change. Given a *q*-value ≤ 0.05, we identified 19 that were upregulated by >1.5 fold (log_2_ FC ≥ 0.6) and 19 that were downregulated by >1.5 fold (log_2_FC ≤ -0.6). These miRNAs are listed in Table 3.

### 3.3. Biological Function Analysis

To identify biological functions involving the DEGs and DE miRNAs, we used ingenuity pathway analysis (IPA). For the 166 DEGs, we found the most impacted, biological functions involved cellular movement, tissue development, cardiovascular system development and function, cellular assembly and organization, and cellular function and maintenance (Table 4). Other functions implicated to a lesser extent were those involving metabolic processes and signal transduction. Similarly, many of the 108 DE miRNAs were predicted to be involved in functions involved in cell movement, survival, development, growth, cardiovascular system development and function, signaling, and organization (Table 5).

### 3.4. qRT-PCR Validation

**Figure 4:**
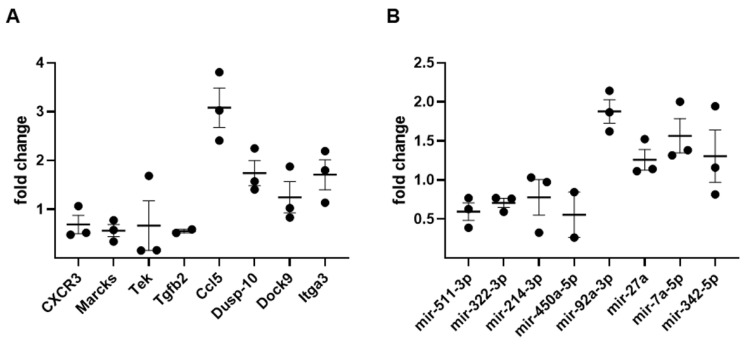
rtPCR confirmation. Illustrated are the fold changes for selected, differentially expressed mRNAs (**A**) or miRNAs (**B**) as determined by qRT-PCR. Downregulated RNAs have an average fold change <1, while upregulated RNAs have a fold change >1.

**Figure 5:**
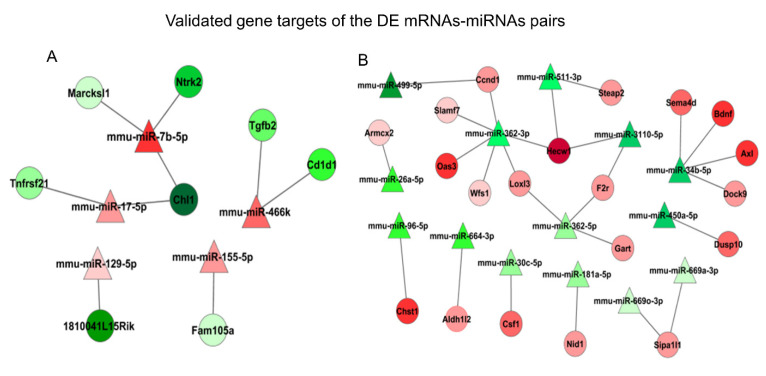
mRNA–miRNA relationships. Illustrated are documented, inverse mRNA–miRNA relationships consistent with the changes in EPCs we identified. (**A**) Downregulated mRNAs (green) and upregulated miRNAs (red); (**B**) upregulated mRNAs (red) and downregulated miRNAs (green). Color shade (darkest to lightest) is indicative of relative FC (greatest to least).

To validate results from our transcriptomics analysis, we performed qRT-PCR for a subset of differentially expressed mRNAs (Figure 4, Table 6) that are involved in those functions identified in the IPA analysis (Table 4). We also performed a similar qRT-PCR analysis of selected DE miRNAs (Figure 4, Table 6). Our results both confirmed the direction of change (upregulated vs. downregulated) and had a high level of consistency with regards to the extent of the change (fold change). Since there is a reciprocal relationship between miRNA expression and the expression of their target mRNAs, we sought to confirm some of these relationships among our DEGs and DE-miRNAs. Consistent with prior reports [29,30,31,32], our RNA-seq data and our qRT-PCR results confirmed reciprocal relationships between mir-214-3p and *Ccl5*, mir-450a-5p and *Dusp-10*, and mir-92a-3p and *Tgfb2* (Figure 4, Table 7). Finally, we used a bioinformatics database [30] to identify additional, documented, reciprocal relationships between the 166 DEGs and the 108 DE miRNAs (Figure 5). Our detected changes in the levels of mir-7b-5p, mir-466k, mir-499-5p, mir-11-3p, mir-3110-5p, mir-34b-5p, and mir-450a-5p (Table 3) were consistent with the inverse expression levels of some target mRNAs, further supporting the validity of our data.

## 4. Discussion

Exposure to PM_2.5_ is associated with cardiovascular morbidity and mortality in humans [2,3] and promotes pre-clinical vascular disease in animal models of exposure [33,34,35]. Some studies propose that quantitative [16,17,36,37] or qualitative [18] defects in EPCs may underlie these outcomes. Yet the mechanistic basis for PM_2.5_-mediated EPC depletion or dysfunction is not completely understood. To this end, in this study, we used a transcriptomic approach to identify gene expression changes in EPCs isolated from the bone marrow of mice exposed to CAP. Compared with control cells isolated from mice breathing filtered air, we identified multiple mRNAs and miRNAs that were either upregulated or downregulated in exposed mice. The top predicted biological functions impacted by these changes are those involving cell movement, cell and tissue development, cell death and survival, and cellular assembly and organization. These predictions are consistent with the role of EPCs in vascular maintenance and repair, and with previously identified, CAP-induced impairments in proliferation, in vitro tube formation, and angiogenesis [18]. Thus, these PM_2.5_-induced changes in EPC gene expression may form the basis of EPC defects and be early indices of incipient cardiovascular disease.

Multiple mechanisms may contribute to the gene expression changes we observed. Epigenetic modifications are implicated, given that the RNA samples used for transcriptomics analysis were isolated from EPCs after 10 days of culture following bone marrow collection from exposed mice. One possible epigenetic mechanism is the post-transcriptional regulation of mRNA levels through targeting miRNAs. Consistent with this idea, we did identify reciprocal relationships between representative dysregulated genes and at least one of their documented, targeting miRNAs (Table 7). Supporting this mechanism of PM_2.5_-induced gene expression changes, other studies also suggested that particle inhalation [38,39] or exposure to particles in cell culture models [40,41] induces the upregulation or downregulation of specific miRNAs. Other epigenetic mechanisms may involve the modification of nuclear material. Methylation of cytosine residues in DNA promoter regions generally inhibits gene expression [42]. In support of this mechanism, some prior studies have determined that exposure to PM_2.5_ induces site-specific DNA methylation, thereby limiting mRNA and protein expression [43,44,45]. DNA methylation is catalyzed by the family of DNA methyltransferases (DNMTs) and, consistently, prior evidence also suggested that PM_2.5_ exposure can activate DNMTs [46]. Furthermore, it has also been shown that reactive oxygen species (ROS) can upregulate DNMT expression [47]. As ROS production is an early and robust outcome of PM_2.5_ inhalation [48], such exposures can thus effectively regulate DNMT expression or activity in promoting methylation. The regulation of gene expression through promoter DNA methylation may be a common outcome of exposure to other environmental toxins as well, and has also been reported following exposure to cigarette smoke [49], volatiles [50], or metals [51], which can also be a constituent of air pollution particles. Finally, in addition to DNA modifications, histone modifications (e.g., methylation, acetylation) can alter chromatin structure, thereby facilitating or impairing transcriptional processes [42]. Recent evidence likewise suggests PM_2.5_ may promote histone acetylation and that this may be accomplished in a dose- and time-dependent manner [52,53].

Exposure to PM_2.5_ is known to negatively impact vascular function and/or the ability of EPCs to maintain vascular homeostasis [18,33,34]. This is supported by our function analysis in the current study, which suggested the influence of PM_2.5_ on genes that regulate cardiovascular system development and function. Furthermore, some of the specific gene expression changes we observed are consistent with PM_2.5_-induced impairments in EPC function. EPC-expressed Cxcr3 is a receptor for multiple CXC chemokines that collectively promote the mobilization, migration, and homing of these cells [54,55]. We found that *Cxcr3* is downregulated in EPCs from CAP-exposed mice (Table 2 and Table 6), thus potentially limiting the availability of these cells for the repair of distal tissue damage. Other gene expression changes we observed are consistent with an impairment in EPC differentiation. For instance, signaling through the angiopoietin receptor Tek (Tie2), a commonly used marker of mature EPCs, is important in controlling the differentiation of these cells [56]. Its downregulation (Table 2 and Table 6) suggests inefficient EPC maturation in exposed animals. Among its effects, the proto-oncogene Myc appears to support cellular de-differentiation and, indeed, was one of the original factors used in the generation of induced pluripotent stem cells [57]. Its upregulation in our study (Supplementary Table S4) further suggests a mechanism whereby PM_2.5_ exposure may impede EPC differentiation. Finally, Ccl5 (Rantes) is a pro-inflammatory cytokine released by early EPCs but to a much lesser extent by late EPCs [58]. The upregulation of *Ccl5* in EPCs derived from CAP-exposed mice (Table 1 and Table 7) is consistent with the idea that these cells remain in an early, immature state, being inefficient in promoting vascular repair. Other DEGs we identified and confirmed by qRT-PCR *(Itga3, Marcks, Dock9, Dusp-10*; Table 6 and Table 7) play general roles in cell adhesion, cytoskeletal organization, growth, and differentiation, and may likewise play essential roles in EPC mobilization, homing, migration, and tissue repair. Some of the DEGs and DE-miRNAs identified in our analysis (Table 1, Table 2 and Table 3) have an uncertain role in EPC function, and a delineation of their roles requires more sophisticated approaches in future studies. Nevertheless, the current work suggests PM_2.5_-induced changes in gene expression may underlie widespread functional defects in EPCs that could lead to impairments in vascular repair and promotion of CVD.

Our findings are significant because they show that PM_2.5_-induced gene expression changes can occur in a tissue (bone marrow) that is distal from the tissue of initial exposure (lungs). These long-range PM_2.5_ effects are believed to initiate largely from redox-catalyzed lipid peroxidation and the generation of toxic intermediates such as aldehydes in the lungs. These intermediates are then distributed systemically through peripheral blood circulation to impact the function of other tissues [59]. There is strong evidence that lipid peroxidation products and aldehydes can influence DNA methylation and histone acetylation patterns [60], as well as miRNA expression levels [61,62]. Thus, PM_2.5_-induced oxidative stress may be causative in the gene expression changes we observed. Supporting this idea, measures taken to limit oxidative stress [18,37], or to neutralize aldehydes [63], appear to limit the downstream, toxic effects of PM_2.5_ exposure and can reverse some of gene expression changes we previously observed [18].

One limitation of this study is that it is not clear if our results are applicable to all exposure scenarios, as PM_2.5_ concentration and composition may vary according to locale and in a seasonal and temporal manner. However, the location of our exposure facility in downtown Louisville assures that particles are derived from diverse and voluminous vehicle exhausts and are generally reflective of traffic-derived PM_2.5_ in any locale. Furthermore, with a 30-day exposure period, we expect the effects of daily variations in composition would be minimized. Consistent with this idea, the predicted biological functions impacted by the gene and miRNA expression changes we observed are consistent with air pollution-induced quantitative and qualitative defects in EPCs that have been previously reported [18,36,37].

## 5. Conclusions

In summary, we identified multiple changes in the expression of distinct mRNAs and miRNAs in bone marrow-derived EPCs isolated from mice inhaling CAP. Some of the predicted biological functions impacted by these changes are those regulating EPC availability or vascular repair potential. These results shed further mechanistic insight with regards to the origin of vascular pathologies associated with air pollution exposures in humans and add to the growing literature suggesting that environmental exposure to volatiles, metals, and chemicals impact health by inducing gene expression changes in diverse cell and tissue types [64,65,66,67,68].

## Figures and Tables

**Table 1:** Upregulated genes.

Ensembl ID	Gene Symbol (Description)	Log_2_(FC)	*q*-Value
ENSMUSG00000090362	Vmn2r79 (vomeronasal 2, receptor 79)	5.8	3.5 × 10^−3^
ENSMUSG00000067855	Speer3 (spermatogenesis associated glutamate (E)-rich protein 3)	5.8	1.1 × 10^−2^
ENSMUSG00000062342	Serpinb9e (serine (or cysteine) peptidase inhibitor, clade B, member 9e)	4.0	3.6 × 10^−2^
ENSMUSG00000020950	Foxg1 (forkhead box G1)	3.2	3.8 × 10^−3^
ENSMUSG00000020911	Krt19 (keratin 19)	3.1	1.2 × 10^−6^
ENSMUSG00000051726	Kcnf1 (potassium voltage-gated channel, subfamily F, member 1)	3.0	1.9 × 10^−2^
ENSMUSG00000021403	Serpinb9b (serine (or cysteine) peptidase inhibitor, clade B, member 9b)	2.9	1.3 × 10^−5^
ENSMUSG00000079654	Prrt4 (proline-rich transmembrane protein 4)	2.8	6.7 × 10^−3^
ENSMUSG00000021301	Hecw1 (HECT, C2, and WW domain-containing E3 ubiquitin protein ligase 1)	2.8	4.1 × 10^−2^
ENSMUSG00000029370	Rassf6 (Ras association (RalGDS/AF-6) domain family member 6)	2.7	2.4 × 10^−2^
ENSMUSG00000035042	Ccl5 (chemokine (C-C motif) ligand 5)	2.5	4.8 × 10^−5^
ENSMUSG00000054855	Rnd1 (Rho family GTPase 1)	2.5	6.3 × 10^−3^
ENSMUSG00000026068	Il18rap (interleukin 18 receptor accessory protein)	2.3	1.9 × 10^−2^
ENSMUSG00000001864	Aif1l (allograft inflammatory factor 1-like)	2.1	1.0 × 10^−2^
ENSMUSG00000032735	Ablim3 (actin binding LIM protein family, member 3)	2.1	1.2 × 10^−2^
ENSMUSG00000020953	Coch (cochlin)	1.9	2.4 × 10^−2^
ENSMUSG00000037185	Krt80 (keratin 80)	1.9	2.8 × 10^−2^
ENSMUSG00000030468	Siglecg (sialic acid binding Ig-like lectin G)	1.9	3.6 × 10^−2^
ENSMUSG00000027313	Chac1 (ChaC, cation transport regulator 1)	1.7	1.4 × 10^−2^
ENSMUSG00000079852	Klra4 (killer cell lectin-like receptor, subfamily A, member 4)	1.7	3.4 × 10^−2^

Listed are the top 20 most upregulated genes (*q* ≤ 0.05; log_2_FC ≥ 0.6), sorted by log_2_FC.

**Table 2:** Downregulated genes.

Ensembl ID	Gene Symbol (Description)	Log_2_(FC)	*q*-Value
ENSMUSG00000030077	Chl1 (cell adhesion molecule L1-like)	−6.2	2.4 × 10^−2^
ENSMUSG00000062760	1810041L15Rik (RIKEN cDNA 1810041L15 gene)	−6.2	3.1 × 10^−2^
ENSMUSG00000045515	Pou3f3 (POU domain, class 3, transcription factor 3)	−6.0	2.1 × 10^−2^
ENSMUSG00000031636	Pdlim3 (PDZ and LIM domain 3)	−5.8	4.9 × 10^−2^
ENSMUSG00000045008	9030612E09Rik (RIKEN cDNA 9030612E09 gene)	−5.7	4.6 × 10^−2^
ENSMUSG00000006386	Tek (TEK receptor tyrosine kinase)	−5.3	1.8 × 10^−2^
ENSMUSG00000022803	Popdc2 (popeye domain containing 2)	−5.2	4.5 × 10^−2^
ENSMUSG00000051242	Pcdhb9 (protocadherin β 9)	−4.7	4.1 × 10^−2^
ENSMUSG00000050493	Fam167b (family with sequence similarity 167, member B)	−3.6	2.0 × 10^−2^
ENSMUSG00000036502	Tmem255a (transmembrane protein 255A)	−3.3	4.5 × 10^−2^
ENSMUSG00000066113	Adamtsl1 (ADAMTS-like 1)	−2.7	2.7 × 10^−2^
ENSMUSG00000020566	Atp6v1c2 (ATPase, H+ transporting, lysosomal V1 subunit C2)	−2.6	1.6 × 10^−4^
ENSMUSG00000038570	Saxo2 (stabilizer of axonemal microtubules 2)	−2.5	1.7 × 10^−2^
ENSMUSG00000055254	Ntrk2 (neurotrophic tyrosine kinase, receptor, type 2)	−2.3	5.0 × 10^−2^
ENSMUSG00000000739	Sult5a1 (sulfotransferase family 5A, member 1)	−2.3	3.7 × 10^−2^
ENSMUSG00000022623	Shank3 (SH3 and multiple ankyrin repeat domains 3)	−1.9	3.8 × 10^−2^
ENSMUSG00000022015	Tnfsf11 (tumor necrosis factor (ligand) superfamily, member 11)	−1.9	3.2 × 10^−2^
ENSMUSG00000027408	Cpxm1 (carboxypeptidase X 1 (M14 family))	−1.7	2.4 × 10^−2^
ENSMUSG00000050232	Cxcr3 (chemokine (C-X-C motif) receptor 3)	−1.3	3.7 × 10^−2^
ENSMUSG00000053414	Hunk (hormonally upregulated Neu-associated kinase)	−1.2	1.8 × 10^−2^

Listed are the top 20 most downregulated genes (*q* ≤ 0.05; log_2_FC < -0.6), sorted by log_2_FC.

**Table 3:** Upregulated and downregulated miRNAs.

Upregulated			Downregulated		
miRNA	FC (log_2_)	*q* Value	miRNA	FC (log_2_)	*q* Value
mmu-miR-6937-5p	3.2	1.8 × 10^−4^	mmu-miR-1899	−2.3	6.8 × 10^−4^
mmu-miR-6399	2.5	2.5 × 10^−4^	mmu-miR-3106-5p	−1.7	1.6 × 10^−4^
mmu-miR-6539	2.1	1.9 × 10^−3^	mmu-miR-486a-3p	−1.6	3.4 × 10^−2^
mmu-miR-344d-3p	1.3	5.7 × 10^−3^	mmu-miR-7649-3p	−1.6	3.6 × 10^−2^
mmu-miR-7043-3p	1.2	5.8 × 10^−6^	mmu-miR-3969	−1.4	2.9 × 10^−3^
mmu-miR-23a-5p	1.2	2.9 × 10^−4^	mmu-miR-708-5p	−1.2	7.0 × 10^−4^
mmu-miR-27a-5p	1.0	6.6 × 10^−3^	mmu-miR-708-3p	−1.1	2.3 × 10^−2^
mmu-miR-7b-5p	0.9	1.8 × 10^−4^	mmu-miR-499-5p	−1.1	2.3 × 10^−2^
mmu-miR-365-2-5p	0.9	1.9 × 10^−3^	mmu-miR-214-3p	−1.0	8.2 × 10^−5^
mmu-miR-376b-3p	0.9	2.6 × 10^−3^	mmu-miR-5619-5p	−1.0	2.1 × 10^−2^
mmu-miR-3473a	0.9	1.1 × 10^−3^	mmu-miR-34b-5p	−0.9	9.9 × 10^−6^
mmu-miR-7a-5p	0.8	1.2 × 10^−10^	mmu-miR-3110-5p	−0.9	2.6 × 10^−2^
mmu-miR-342-5p	0.8	1.8 × 10^−4^	mmu-miR-450a-5p	−0.8	1.1 × 10^−4^
mmu-miR-7654-5p	0.7	4.8 × 10^−3^	mmu-miR-6239	−0.8	4.7 × 10^−5^
mmu-miR-466h-5p	0.7	3.0 × 10^−4^	mmu-miR-450a-2-3p	−0.8	3.8 × 10^−3^
mmu-miR-92a-3p	0.7	6.8 × 10^−3^	mmu-miR-700-5p	−0.7	4.6 × 10^−2^
mmu-miR-200c-3p	0.6	3.5 × 10^−3^	mmu-miR-188-3p	−0.7	2.1 × 10^−3^
mmu-miR-466k	0.6	1.0 × 10^−4^	mmu-miR-511-3p	−0.7	4.3 × 10^−3^
mmu-miR-30d-3p	0.6	1.9 × 10^−6^	mmu-miR-322-3p	−0.6	2.8 × 10^−3^
mmu-miR-877-3p	0.6	1.4 × 10^−3^			

Listed are the most upregulated and downregulated miRNAs (*q* ≤ 0.05; upregulated: log_2_FC ≥ 0.6; downregulated: log_2_FC < −0.6), sorted by log_2_FC.

**Table 4:** Biological functions—mRNAs.

Category	*p*-Value	Number	Representative Genes
Cellular movement	1.53 × 10^−10^–2.52 × 10^−4^	56	Ccl5, Foxg1, Itga3, Krt19, Marcks, Tek, Tgfb2, Tnsf11
Tissue development	4.43 × 10^−10^–2.33 × 10^−4^	66	Ccl5, Dusp-10, Foxg1, Itga3, Krt19, Marcks, Myc, Ntrk2, Tnfsf11
Cardiovascular system development and function	1.42 × 10^−9^–2.5 × 10^−4^	37	Ccl5, Cxcr3, Itga3, Myc, Ntrk2, Rnd1, Tek, Tgfb2, Tnsf11
Cellular assembly and organization	1.03 × 10^−8^–1.94 × 10^−4^	43	Ccl5, Cxcr3, Itga3, Krt19, Myc, Ntrk2, Pdlim3, Rnd1, Tek, Tgfb2, Tnfsf11
Cellular function and maintenance	1.03 × 10^−8^–1.94 × 10^−4^	39	Ablim3, Ccl5, Cxcr3, Itga3, Myc, Ntrk2, Rnd1, Tek, Tgfb2, Tnfsf11
Cellular development	1.44 × 10^−7^–1.99 × 10^−4^	52	Ccl5, Chl1, Cxcr3, Dusp10, Foxg1, Itga3, Marcks, Myc, Ntrk2, Tek, Tgfb2, Tnfsf11
Cellular growth and proliferation	1.44 × 10^−7^–2.04 × 10^−4^	61	Ccl5, Chl1, Cxcr3, Dusp-10, Foxg1, Il18rap, Itga3, Marcks, Myc, Ntrk2, Tek, Tgfb2, Tnfsf11
Cell death and survival	2.53 × 10^−7^–2.39 × 10^−4^	56	Ccl5, Chl1, Cxcr3, Dusp-10, Foxg1, Itga3, Myc, Ntrk2, Pou3f3, Tek, Tgfb2, Tnfsf11
Connective tissue disorders	3.02 × 10^−7^–1.99 × 10^−4^	43	Adamtsl1, Ccl5, Cxcr3, Dusp-10, Ntrk2, Tgfb2, Tnfsf11
Cell-to-cell signaling and interaction	3.38 × 10^−7^–2.47 × 10^−4^	31	Ccl5, Cxcr3, Itga3, Ntrk2, Tek, Tgfb2, Tnfsf11
Cell cycle	6.15 × 10^−7^–2.29 × 10^−4^	19	Ccl5, Foxg1, Myc, Ntrk2, Tnfsf11
Tissue morphology	8.46 × 10^−7^–1.89 × 10^−4^	53	Ccl5, Cxcr3, Dusp-10, Itga3, Krt19, Myc, Ntrk2, Tek, Tgfb2, Tnfsf11
Cell morphology	2.67 × 10^−6^–1.89 × 10^−4^	38	Ablim3, Ccl5, Chl1, Cxcr3, Foxg1, Itga3, Myc, Ntrk2, Pou3f3, Tek, Tgfb2, Tnfsf11
Post-translational modification	2.73 × 10^−6^–2.46 × 10^−4^	20	Ccl5, Ntrk2, Tek, Tgfb2, Tnfsf11
Connective tissue development and function	1.24 × 10^−5^–1.89 × 10^−4^	17	Cxcr3, Myc, Tgfb2, Tnfsf11
DNA replication, recombination, and repair	2.22 × 10^−5^–1.52 × 10^−4^	15	Ccl5, Cxcr3, Myc, Ntrk2, Tgfb2, Tnfsf11
Hematopoiesis	2.41 × 10^−5^–1.68 × 10^−4^	29	Ccl5, Cxcr3, Dusp-10, Marcks, Myc, Tek, Tnfsf11
Amino acid metabolism	≤9.78 × 10^−5^	3	Myc
Molecular transport	9.78 × 10^−5^–2.29 × 10^−4^	15	Ccl5, Cxcr3, Myc, Tnfsf11
Small molecule biochemistry	9.78 × 10^−5^–2.29 × 10^−4^	6	Myc, Tnfsf11
Energy production	≤2.29 × 10^−4^	4	Tnfsf11
Nucleic acid metabolism	≤2.29 × 10^−4^	4	Tnfsf11
Cell signaling	≤2.46 × 10^−4^	7	Ccl5

Listed are the top biological functions impacted by the mRNA changes we detected, their significance, the number of DEGs that impact these functions, and representative DEGs as listed in Supplementary Tables S4 and S5. Selected genes are boxed in Figure 2B.

**Table 5:** Biological functions—miRNAs.

Category	*p*-Value	Number	Representative miRNA
Cellular movement	1.18 × 10^−6^–4.59 × 10^−2^	16	mir-7a-5p, mir-92a-3p
Cell death and survival	1.71 × 10^−6^–4.59 × 10^−2^	16	mir-214-3p
Cellular development	3.45 × 10^−5^–4.15 × 10^−2^	21	mir-27a-3p, mir-92a-3p
Cellular growth and proliferation	3.45 × 10^−5^–3.65 × 10^−2^	16	mir-27a-3p, mir-92a-3p
Cardiovascular system development and function	1.95 × 10^−4^–3.9 × 10^−2^	14	mir-27a-3p, mir-214-3p, mir-486-3p, mir-34a-5p
Cell cycle	3.61 × 10^−4^–4.48 × 10^−2^	6	mir-27a-3p
Cell morphology	5.51 × 10^−3^–3.9 × 10^−2^	5	mir-214-3p
Cell-to-cell signaling and interaction	1.37 × 10^−2^–2.46 × 10^−2^	5	mir-34a-5p
Cellular assembly and organization	1.37 × 10^−2^–4.85 × 10^−2^	3	mir-708-5p
Cellular function and maintenance	1.37 × 10^−2^–3.53 × 10^−2^	6	mir-34a-5p

Listed are the top 10 biological functions impacted by the miRNA changes we detected, their significance, the number of DE miRNAs that impact these functions, and representative miRNAs as listed in Table 3. Selected miRNAs are boxed in Figure 3B.

**Table 6:** Confirmation of RNA-seq results.

**DEG**	**FC-RNA-Seq**	**FC–qRT-PCR (SE)**
Cxcr3	0.406	0.687 (0.192)
Marcks	0.550	0.563 (0.128)
Tek	0.025	0.666 (0.517)
Dock9	1.66	1.24 (0.330)
Itga3	2.07	1.71 (0.313)
**DE miRNA**	**FC-RNA-Seq**	**FC–qRT-PCR (SE)**
mir-511-3p	0.630	0.595 (0.113)
mir-322-3p	0.64	0.707 (0.059)
mir-27a	2.28	1.26 (0.134)
mir-342-5p	1.69	1.31 (0.340)
mir-7a-5p	1.79	1.57 (0.222)

Listed are representative DEGs or DE miRNAs and their fold changes (FC), as determined by RNA-seq analysis and qRT-PCR (*n* = 9).

**Table 7:** Confirmation of reciprocal mRNA–miRNA relationships.

Representative Gene			Representative Reciprocal miRNA		
Gene	FC-RNA-seq	FC–qRT-PCR (SE)	miRNA	FC-RNA-Seq	FC-qRT-PCR (SE)
Ccl5	5.75	3.08 (0.412)	mir-214-3p	0.493	0.778 (0.226)
Dusp-10	1.81	1.74 (0.26)	mir-450a-5p	0.555	0.560 (0.241)
Tgfb2	0.535	0.551 (0.029)	mir-92a-3p	1.58	1.88 (0.15)

Listed are representative DEGs, a representative targeting DE miRNA, and their respective fold changes (FC) as determined by RNA-seq analysis and qRT-PCR (*n* = 9 samples).

## Data Availability

The sequence datasets supporting the conclusions of this article are available in the Gene Expression Omnibus (GEO) repository (https://www.ncbi.nlm.nih.gov/geo/, accessed on 24 June 2020; accession number: GSE153038).

## Supplementary Materials

The following are available online at https://www.mdpi.com/article/10.3390/genes12071058/s1, Table S1. Summary of initial sequence analysis (mRNA). Table S2. Summary of initial sequence analysis (miRNA). Table S3. List of rtPCR primers used. Table S4. Upregulated genes. Listed are upregulated genes (*q* < 0.05; log_2_FC ≥ 0.6). Table S5. Downregulated genes. Listed are downregulated genes (*q* < 0.05; |log_2_FC| > 0.66).

## Institutional Animal Care and Use Committee

All animal procedures were approved by the University of Louisville Institutional Animal Care and Use Committee (#14035).
