# Supplementary material for: Exposure to Fine Particulate Matter Air Pollution Alters mRNA and miRNA Expression in Bone Marrow-Derived Endothelial Progenitor Cells from Mice
Source: Genes (Basel). 2021 Jul 10;12(7):1058. doi: 10.3390/genes12071058 (PMC8307414; doi:10.3390/genes12071058)
Supplement: Supplementary file 1 [file genes-12-01058-s001.zip › genes-1258575-supplementary.pdf]

GCAT TACG GCAT genes

MDPI

SUPPORTING INFORMATION

Table S1. Summary of initial sequence analysis (mRNA).

Figure	Input Reads	Aligned Reads	Alignment Rate
Air_1-282934788	35,276,282	34,922,845	98.99%
Air_2-282924076	35,968,212	35,648,988	99.11%
Air_3-282937791	34,981,048	34,676,970	99.13%
CAPs_1-282924078	36,935,481	36,589,704	99.06%
CAPs_2-282937793	35,278,053	34,953,185	99.07%
CAPs_3-282941822	35,485,165	35,149,979	99.05%

Table S2. Summary of initial sequence analysis (miRNA).

File	Input Reads	After Trimming	Aligned Reads	Alignment Rate
miR-01-Air-1_S1_ALL_R1	28,324,999	27,387,531	23,330,772	85.19%
miR-02-Air-2_S2_ALL_R1	25,980,741	25,034,793	22,632,478	90.40%
miR-03-Air-3_S3_ALL_R1	24,917,052	23,918,015	21,973,143	91.87%
miR-04-CAPs-1_S8_ALL_R1	27,173,935	25,655,665	21,536,485	83.94%
miR-05-CAPs-2_S9_ALL_R1	27,944,476	26,790,331	25,532,775	95.31%
miR-06-CAPs-3_S10_ALL_R1	20,799,737	20,287,182	19,978,586	98.48%

Table S3. List of rtPCR primers.

DEG	Primer Assay ID #
Cxcr3	Mm99999054_s1
Marcks	Mm025243303_s1
Tek	Mm00443243_m1
Dock9	Mm01224565_m1
Itga3	Mm00442910_m1
Ccl5	Mm01302427_m1
Dusp-10	Mm00517678_m1
Tgfb2	Mm00436955_m1
**DE miRNA**	**Primer Assay ID #**
mir-511-3p	463069_mat
mir-322-3p	002506
mir-27a	002445
mir-342-5p	002527
mir-7a-5p	000268
mir-214-3p	002306
mir-450a-5p	002303
mir-92a-3p	000430

Table S4. Up-regulated genes ($q \leq 0.05$; $\log_2 FC \geq 0.6$).

Ensembl ID	Gene symbol (Description)	$\log_2$(FC)	*q* value
ENSMUSG00000090362	Vmn2r79 (vomeronasal 2, receptor 79)	5.84	3.5×10^{-3}

ENSMUSG00000067855	Speer3 (spermatogenesis associated glutamate (E)-rich protein 3)	5.84	1.1×10^{-2}
ENSMUSG00000062342	Serpinb9e (serine (or cysteine) peptidase inhibitor, clade B, member 9e)	4.00	3.6×10^{-2}
ENSMUSG00000020950	Foxg1 (forkhead box G1)	3.21	3.8×10^{-3}
ENSMUSG00000020911	Krt19 (keratin 19)	3.10	1.2×10^{-6}
ENSMUSG00000051726	Kcnf1 (potassium voltage-gated channel, subfamily F, member 1)	2.99	1.9×10^{-2}
ENSMUSG00000021403	Serpinb9b (serine (or cysteine) peptidase inhibitor, clade B, member 9b)	2.90	1.3×10^{-5}
ENSMUSG00000079654	Prrt4 (proline-rich transmembrane protein 4)	2.82	6.7×10^{-3}
ENSMUSG00000021301	Hecw1 (HECT, C2 and WW domain containing E3 ubiquitin protein ligase 1)	2.81	4.1×10^{-2}
ENSMUSG00000029370	Rassf6 (Ras association (RalGDS/AF-6) domain family member 6)	2.68	2.4×10^{-2}
ENSMUSG00000035042	Ccl5 (chemokine (C-C motif) ligand 5)	2.52	4.8×10^{-5}
ENSMUSG00000054855	Rnd1 (Rho family GTPase 1)	2.49	6.3×10^{-3}
ENSMUSG00000026068	Il18rap (interleukin 18 receptor accessory protein)	2.33	1.9×10^{-2}
ENSMUSG00000001864	Aif1l (allograft inflammatory factor 1-like)	2.15	1.0×10^{-2}
ENSMUSG00000032735	Ablim3 (actin binding LIM protein family, member 3)	2.15	1.2×10^{-2}
ENSMUSG00000020953	Coch (cochlin)	1.90	2.4×10^{-2}
ENSMUSG00000037185	Krt80 (keratin 80)	1.89	2.8×10^{-2}
ENSMUSG00000030468	Siglecg (sialic acid binding Ig-like lectin G)	1.85	3.6×10^{-2}
ENSMUSG00000027313	Chac1 (ChaC, cation transport regulator 1)	1.73	1.4×10^{-2}
ENSMUSG00000079852	Klra4 (killer cell lectin-like receptor, subfamily A, member 4)	1.72	3.4×10^{-2}
ENSMUSG00000022157	Mcpt8 (mast cell protease 8)	1.64	1.86×10^{-2}
ENSMUSG00000048482	Bdnf (brain derived neurotrophic factor)	1.58	4.36×10^{-2}
ENSMUSG00000021485	Mxd3 (Max dimerization protein 3)	1.56	4.13×10^{-2}
ENSMUSG00000046733	Gprc5a (G protein-coupled receptor, family C, group 5, member A)	1.55	4.40×10^{-2}
ENSMUSG00000030353	Tead4 (TEA domain family member 4)	1.42	3.32×10^{-3}
ENSMUSG00000099703	Gm28285 (predicted gene 28285)	1.40	2.16×10^{-2}
ENSMUSG00000027068	Dhrs9 (dehydrogenase/reductase (SDR family) member)	1.30	1.42×10^{-2}
ENSMUSG00000003849	Nqo1 (NAD(P)H dehydrogenase, quinone 1)	1.26	9.19×10^{-3}
ENSMUSG00000006398	Cdc20 (cell division cycle 20)	1.26	1.04×10^{-2}
ENSMUSG00000040675	Mthfd1l (methylenetetrahydrofolate dehydrogenase (NADP+ dependent) 1-like)	1.16	6.19×10^{-3}
ENSMUSG00000032661	Oas3 (2'-5' oligoadenylate synthetase 3)	1.14	6.07×10^{-14}
ENSMUSG00000025348	Itga7 (integrin alpha 7)	1.13	4.40×10^{-2}
ENSMUSG00000003779	Kif20a (kinesin family member 20A)	1.13	1.11×10^{-2}
ENSMUSG00000029414	Kntc1 (kinetochore associated 1)	1.11	1.69×10^{-2}
ENSMUSG00000040010	Slc7a5 (solute carrier family 7 (cationic amino acid transporter, y+ system), member 5)	1.11	2.39×10^{-2}
ENSMUSG00000037725	Ckap2 (cytoskeleton associated protein 2)	1.10	9.14×10^{-3}
ENSMUSG00000032815	Fanca (Fanconi anemia, complementation group A)	1.10	2.62×10^{-2}
ENSMUSG00000015854	Cd5l (CD5 antigen-like)	1.10	1.96×10^{-8}
ENSMUSG00000027221	Chst1 (carbohydrate (keratan sulfate Gal-6) sulfotransferase 1)	1.09	2.16×10^{-2}
ENSMUSG00000025185	Loxl4 (lysyl oxidase-like 4)	1.07	3.35×10^{-2}

ENSMUSG00000001507	Itga3 (integrin alpha 3)	1.05	1.11×10^{-3}
ENSMUSG00000003355	Fkbp11 (FK506 binding protein 11)	1.04	6.65×10^{-3}
ENSMUSG00000020089	Ppa1 (pyrophosphatase (inorganic) 1)	1.03	1.99×10^{-2}
ENSMUSG00000021214	Akr1c18 (aldo-keto reductase family 1, member C18)	1.02	3.11×10^{-2}
ENSMUSG00000030867	Plk1 (polo like kinase 1)	1.00	3.62×10^{-2}
ENSMUSG00000006442	Srm (spermidine synthase)	0.99	3.56×10^{-2}
ENSMUSG00000002602	Axl (AXL receptor tyrosine kinase)	0.99	1.32×10^{-2}
ENSMUSG00000035107	Dcbld2 (discoidin, CUB and LCCL domain containing 2)	0.98	2.06×10^{-2}
ENSMUSG00000042606	Hirip3 (HIRA interacting protein 3)	0.97	3.56×10^{-2}
ENSMUSG00000042793	Lgr6 (leucine-rich repeat-containing G protein-coupled receptor 6)	0.96	2.57×10^{-3}
ENSMUSG00000013629	Cad (carbamoyl-phosphate synthetase 2, aspartate transcarbamylase, and dihydroorotase)	0.94	3.56×10^{-2}
ENSMUSG00000022346	Myc (myelocytomatosis oncogene)	0.94	4.71×10^{-2}
ENSMUSG00000021270	Hsp90aa1 (heat shock protein 90, alpha (cytosolic), class A member 1)	0.93	3.04×10^{-2}
ENSMUSG00000030878	Cdr2 (cerebellar degeneration-related 2)	0.93	1.52×10^{-2}
ENSMUSG00000027469	Tpx2 (TPX2, microtubule-associated)	0.91	4.40×10^{-2}
ENSMUSG00000021451	Sema4d (sema domain, immunoglobulin domain (Ig), transmembrane domain (TM) and short cytoplasmic domain, (semaphorin) 4D)	0.91	1.49×10^{-2}
ENSMUSG00000028069	Gpatch4(G patch domain containing 4)	0.89	2.07×10^{-2}
ENSMUSG00000020256	Aldh1l2 (aldehyde dehydrogenase 1 family, member L2)	0.88	2.40×10^{-2}
ENSMUSG00000037946	Fgd3 (FYVE, RhoGEF and PH domain containing 3)	0.88	1.52×10^{-2}
ENSMUSG00000004655	Aqp1 (aquaporin 1)	0.85	2.69×10^{-2}
ENSMUSG00000039384	Dusp10 (dual specificity phosphatase 10)	0.85	4.96×10^{-2}
ENSMUSG00000076437	Selenoh (selenoprotein H)	0.84	2.37×10^{-2}
ENSMUSG00000031821	Gins2 (GINS complex subunit 2 (Psf2 homolog))	0.83	1.86×10^{-2}
ENSMUSG00000032548	Slco2a1 (solute carrier organic anion transporter family, member 2a1)	0.83	3.57×10^{-2}
ENSMUSG00000055044	Pdlim1 (PDZ and LIM domain 1 (elfin))	0.82	2.62×10^{-2}
ENSMUSG00000025403	Shmt2 (serine hydroxymethyltransferase 2 (mitochondrial))	0.81	4.89×10^{-2}
ENSMUSG00000014599	Csf1 (colony stimulating factor 1 (macrophage))	0.80	3.39×10^{-2}
ENSMUSG00000026042	Col5a2 (collagen, type V, alpha 2)	0.79	9.14×10^{-3}
ENSMUSG00000026558	Uck2 (uridine-cytidine kinase 2)	0.79	4.89×10^{-2}
ENSMUSG00000070348	Ccnd1 (cyclin D1)	0.78	4.09×10^{-2}
ENSMUSG00000032690	Oas2 (2'-5' oligoadenylate synthetase 2)	0.77	1.91×10^{-3}
ENSMUSG00000037907	Ankrd13b (ankyrin repeat domain 13b)	0.77	3.60×10^{-2}
ENSMUSG00000028883	Sema3a (sema domain, immunoglobulin domain (Ig), short basic domain, secreted, (semaphorin) 3A)	0.77	9.27×10^{-3}
ENSMUSG00000051065	Mb21d2∣Mab-21 domain containing 2)	0.76	4.85×10^{-2}
ENSMUSG00000029752	Asns (asparagine synthetase)	0.76	3.68×10^{-2}
ENSMUSG00000072235	Tuba1a (tubulin, alpha 1A)	0.75	4.85×10^{-2}
ENSMUSG00000000693	Loxl3 (lysyl oxidase-like 3)	0.74	6.54×10^{-3}
ENSMUSG00000022962	Gart (phosphoribosylglycinamide formyltransferase)	0.74	2.43×10^{-2}
ENSMUSG00000025558	Dock9 (dedicator of cytokinesis 9)	0.74	3.08×10^{-2}
ENSMUSG00000033788	Dysf (dysferlin)	0.73	2.39×10^{-2}
ENSMUSG00000001131	Timp (tissue inhibitor of metalloproteinase 1)	0.72	1.04×10^{-2}

ENSMUSG00000068699	Flnc (filamin C, gamma)	0.71	3.32×10^{-3}
ENSMUSG00000056612	Ppp1r14b (protein phosphatase 1, regulatory (inhibitor) subunit 14B)	0.71	3.60×10^{-2}
ENSMUSG00000030835	Nomo1 (nodal modulator 1)	0.71	1.39×10^{-2}
ENSMUSG00000027580	Helz2 (helicase with zinc finger 2, transcriptional coactivator)	0.71	3.34×10^{-5}
ENSMUSG00000070436	Serpinh1 (serine (or cysteine) peptidase inhibitor, clade H, member 1)	0.71	6.65×10^{-3}
ENSMUSG00000032060	Cryab (crystallin, alpha B)	0.70	1.52×10^{-2}
ENSMUSG00000005397	Nid1 (nidogen 1)	0.70	3.56×10^{-2}
ENSMUSG00000021453	Gadd45g (growth arrest and DNA-damage-inducible 45 gamma)	0.70	6.11×10^{-3}
ENSMUSG00000028776	Tinagl1 (tubulointerstitial nephritis antigen-like 1)	0.69	3.57×10^{-2}
ENSMUSG00000048537	Phldb1 (pleckstrin homology like domain, family B, member 1)	0.67	4.23×10^{-2}
ENSMUSG00000048376	F2r (coagulation factor II (thrombin) receptor)	0.67	3.23×10^{-2}
ENSMUSG00000023800	Tiam2 (T cell lymphoma invasion and metastasis 2)	0.67	4.13×10^{-2}
ENSMUSG00000024613	Tcof1 (treacle ribosome biogenesis factor 1)	0.67	2.81×10^{-2}
ENSMUSG00000064080	Fbln2 (fibulin 2)	0.66	3.32×10^{-3}
ENSMUSG00000036273	Lrrk2 (leucine-rich repeat kinase 2)	0.66	1.22
ENSMUSG00000015947	Fcgr1 (Fc receptor, IgG, high affinity I)	0.65	3.60×10^{-5}
ENSMUSG00000042700	Sipa1l1 (signal-induced proliferation-associated 1 like 1)	0.65	3.87×10^{-3}
ENSMUSG00000037851	Iars (isoleucine-tRNA synthetase)	0.65	4.71×10^{-2}
ENSMUSG00000090958	Lrrc32 (leucine rich repeat containing 32)	0.63	2.39×10^{-2}
ENSMUSG00000025324	Atp10a (ATPase, class V, type 10A)	0.62	3.24×10^{-2}
ENSMUSG00000034903	Cobll1 (Cobl-like 1)	0.62	2.57×10^{-3}
ENSMUSG00000052776	Oas1a (2'-5' oligoadenylate synthetase 1A)	0.60	4.96×10^{-2}
ENSMUSG00000015653	Steap2 (six transmembrane epithelial antigen of prostate 2)	0.60	4.52×10^{-2}

Table S5. Down-regulated genes ($q \leq 0.05$; $\log_2 FC < -0.6$).

Ensembl ID.	Gene symbol (Description)	$\log_2$(FC)	q value
ENSMUSG00000030077	Chl1 (cell adhesion molecule L1-like)	−6.22	2.39×10^{-2}
ENSMUSG00000062760	1810041L15Rik (RIKEN cDNA 1810041L15 gene)	−6.19	3.11×10^{-2}
ENSMUSG00000045515	Pou3f3 (POU domain, class 3, transcription factor 3)	−6.03	2.06×10^{-2}
ENSMUSG00000031636	Pdlim3 (PDZ and LIM domain 3)	−5.81	4.90×10^{-2}
ENSMUSG00000045008	9030612E09Rik (RIKEN cDNA 9030612E09 gene)	−5.71	4.57×10^{-2}
ENSMUSG00000006386	Tek (TEK receptor tyrosine kinase)	−5.28	1.75×10^{-2}
ENSMUSG00000022803	Popdc2 (popeye domain containing 2)	−5.19	4.54×10^{-2}
ENSMUSG00000051242	Pcdhb9 (protocadherin beta 9)	−4.68	4.13×10^{-2}
ENSMUSG00000050493	Fam167b (family with sequence similarity 167, member B)	−3.62	1.99×10^{-2}
ENSMUSG00000036502	Tmem255a (transmembrane protein 255A)	−3.26	4.54×10^{-2}
ENSMUSG00000066113	Adamtsl1 (ADAMTS-like 1)	−2.73	2.69×10^{-2}
ENSMUSG00000020566	Atp6v1c2 (ATPase, H+ transporting, lysosomal V1 subunit C2)	−2.56	1.62×10^{-4}
ENSMUSG00000038570	Saxo2 (stabilizer of axonemal microtubules 2)	−2.46	1.69×10^{-2}
ENSMUSG00000055254	Ntrk2 (neurotrophic tyrosine kinase, receptor, type 2)	−2.34	4.96×10^{-2}
ENSMUSG00000000739	Sult5a1 (sulfotransferase family 5A, member 1)	−2.33	3.67×10^{-2}
ENSMUSG00000022623	Shank3 (SH3 and multiple ankyrin repeat domains 3)	−1.94	3.81×10^{-2}

ENSMUSG00000022015	Tnfsf11(tumor necrosis factor (ligand) superfamily, member 11)	−1.86	3.24×10^{-2}
ENSMUSG00000027408	Cpxm1 (carboxypeptidase X 1 (M14 family))	−1.72	2.39×10^{-2}
ENSMUSG00000050232	Cxcr3(chemokine (C-X-C motif) receptor 3)	−1.26	3.67×10^{-2}
ENSMUSG00000053414	Hunk (hormonally upregulated Neu-associated kinase)	−1.19	1.75×10^{-2}
ENSMUSG00000021536	Adcy2l (denylate cyclase 2)	−1.19	1.86×10^{-2}
ENSMUSG00000028076	Cd1d1 (CD1d1 antigen)	−1.18	1.79×10^{-2}
ENSMUSG00000020017	Hal (histidine ammonia lyase)	−1.02	3.23×10^{-2}
ENSMUSG00000014782	Plekhg4 (pleckstrin homology domain containing, family G (with RhoGef domain) member 4)	−1.02	3.56×10^{-2}
ENSMUSG00000095440	Fignl2 (fidgetin-like 2)	−0.95	3.33×10^{-2}
ENSMUSG00000029373	Pf4 (platelet factor 4)	−0.93	3.68×10^{-2}
ENSMUSG00000039239	Tgfb2 (transforming growth factor, beta 2)	−0.90	4.96×10^{-2}
ENSMUSG00000083282	Ctsf (cathepsin F)	−0.89	4.57×10^{-2}
ENSMUSG00000069662	Marcks (myristoylated alanine rich protein kinase C substrate)	−0.86	4.96×10^{-2}
ENSMUSG00000056185	Snx32 (sorting nexin 32)	−0.85	4.71×10^{-2}
ENSMUSG00000053646	Plxnb (lplexin B1)	−0.82	3.20×10^{-2}
ENSMUSG00000002808	Epdr1(ependymin related protein 1)	−0.77	3.56×10^{-2}
ENSMUSG00000066829	Zfp810 (zinc finger protein 810)	−0.77	4.23×10^{-2}
ENSMUSG00000023915	Tnfrsf21(tumor necrosis factor receptor superfamily, member 21)	−0.74	1.76×10^{-4}
